# Risk Factors for Introduction of H5N1 Highly Pathogenic Avian Influenza Virus in Japanese Commercial Layer Farms During the 2022–2023 Epidemic: A Case–Control Study

**DOI:** 10.1155/tbed/2658633

**Published:** 2025-11-12

**Authors:** Sonoko Kondo, Emi Yamaguchi, Yoko Hayama, Takehisa Yamamoto

**Affiliations:** Epidemiology and Arbovirus Group, Division of Transboundary Animal Disease Research, National Institute of Animal Health, National Agriculture and Food Research Organization, Tsukuba, Ibaraki, Japan

## Abstract

Japan experienced its largest outbreak of highly pathogenic avian influenza (HPAI) during the 2022–2023 season, with 84 cases on poultry farms and the culling of more than 17 million birds. Commercial layer farms were the most affected, with 58 cases caused by the H5N1 subtype of the HPAI virus. To identify the risk factors and support future outbreak preparedness, a case–control study was conducted. All 58 affected commercial layer farms were designated as case farms, whereas 26 unaffected farms located within a 5 km radius of the case farms served as control farms. Data were collected through structured interviews using a questionnaire for the control farms and from on-site investigation records for the case farms. For control farms, all poultry barns on the premises were included, whereas only the initially affected barns were analyzed in the case farms. Logistic regression using a generalized linear mixed model was performed to evaluate the associations between HPAI outbreaks and explanatory variables related to farm- and barn-level factors, such as farm characteristics, husbandry and hygiene practices, barn structure, and environmental factors, including wildlife presence. Farm location was included as a random effect. The analysis identified a large number of laying hens on the farm (≥100,000 birds) as a potential risk factor. In contrast, changing shoes at the barn entrance and changing shoes or avoiding the use of the back door were identified as potential protective factors. These findings provide evidence-based guidance for strengthening biosecurity measures and improving HPAI prevention strategies on poultry farms in Japan.

## 1. Introduction

Highly pathogenic avian influenza (HPAI) is a disease that affects domestic and wild birds and is caused by the HPAI virus (HPAIV). It poses a serious challenge to the global poultry industry because of its high mortality and potential threat to public health [1]. In Japan, HPAI in poultry, caused by the H5 and H7 subtypes of HPAIV, is designated as a notifiable disease by law. When an outbreak occurs, all the poultry on the affected farm are culled to contain the spread of the disease.

HPAI outbreaks have been periodically reported in Japan since the first case was confirmed in 2004, following a 79-year absence of the disease. However, since the winter of 2020, outbreaks have occurred annually, raising serious concerns for the domestic poultry industry and food security. Specifically, the 2022–2023 season marked the largest outbreak to date, with 84 cases reported across 26 prefectures between October 28, 2022, and April 7, 2023. During this period, more than 17 million birds were culled [2]. Of the 84 cases, 83 were caused by the H5N1 subtype of HPAIV, and one was attributed to the H5N2 subtype [3]. Phylogenetic analysis demonstrated that the HPAIV isolated during this season belonged to the G2 group of clade 2.3.4.4b, the ancestral virus that was detected in Europe in late 2020 [4]. During the HPAI outbreaks in the 2022–2023 season, layer farms accounted for the highest number of cases (58), followed by broilers (11), layer growers (3), broiler chicks (1), ducks (7), quails (1), ostriches and emus (2), and guinea fowl (1) [2].

In Japan, all cases of HPAI in domestic poultry farms have been investigated by teams composed of national and prefectural animal health officials and experts. These investigations are typically conducted immediately after an outbreak is confirmed. Interviews were carried out to gather information on the course of the outbreak, assess the surrounding environment, document sightings of wild birds, and evaluate husbandry practices, hygiene management, and biosecurity of poultry housing. The findings were published in an epidemiological investigation report at the end of the epizootic season. A report for the 2022–2023 season indicated that many cases of HPAI occurred on farms located near ponds, rivers, canals, rice paddies, or other water bodies that are frequented by migratory birds. Furthermore, live wild birds, such as crows, and signs of wild animals were frequently observed within the premises of affected farms and nearby wooded areas. Several farms have reported damage to the roofs and walls of poultry houses, which may have served as entry points for wild animals [2]. Regarding poultry housing, approximately half of the HPAI cases on layer farms occurred in closed-type barns, which are generally considered less accessible to wildlife than open-sided housing. This finding raises questions regarding the relationship between housing structures and HPAI risk. In addition, outbreaks at large-scale farms were a prominent feature of the season. Ten outbreaks occurred on layer farms housing more than 500,000 birds, accounting for 54% of all birds culled during the season [2]. As large-scale farms often use closed-type poultry houses, further investigation is needed to determine whether the housing type or farm size contributes more to the risk of infection.

Several studies have identified risk factors associated with HPAI outbreaks on poultry farms. Migratory wild birds, particularly waterfowl, are considered natural hosts of avian influenza viruses, and the long-distance spread of the virus through bird migration has been documented in multiple studies [5–7]. In Japan, migratory birds arriving each autumn from overseas breeding sites, such as Siberia, are believed to introduce the virus into the country [8, 9]. Previous studies have suggested that farm location, type, and size are associated with the risk of HPAI outbreaks in poultry. A case–control study on the H5N8 outbreak in Japan during the 2020–2021 season, using the data from the national farm database, analyzed the relationships between outbreak risk and several environmental factors, including the presence of rice paddies, forests, and nearby poultry farms, the proximity to water bodies, and the number of birds kept. The results indicated a higher risk of outbreaks in layer farms, farms with larger bird populations, and those located closer to water sources. On broiler farms, shorter distances to water bodies were associated with a higher risk of infection [10]. Other risk factors identified in previous studies include bird species and flock size [11], presence of nearby HPAI outbreaks or whether the farm is located within restricted zones [11–13], wild bird population density [14], regional poultry farm density [15], distance from water bodies [16–18], biosecurity measures [12, 19–21], and sightings of wild birds on farms [22].

To identify risk factors associated with HPAI outbreaks, it is essential to compare the characteristics of outbreak and nonoutbreak farms. However, for nonoutbreak farms, only limited information, such as farm type, bird population, and location, was available in the national farm database, preventing meaningful comparisons in terms of husbandry practices, facilities such as barn structures, and biosecurity compliance. To address this limitation, we conducted a case–control study focusing on the 2022–2023 HPAI outbreak by collecting detailed data from both affected and nearby unaffected farms to explore the association between outbreak risk and farm-related factors.

## 2. Materials and Methods

### 2.1. Farm Selection

This study focused on layer farms, which accounted for the highest number of cases during the 2022–2023 HPAI outbreak. All commercial layer farms in which HPAI outbreaks were confirmed between October 2022 and April 2023 were designated as case farms. Control farms were randomly selected from unaffected commercial layer farms located within a 5 km radius of each case farm that agreed to participate in the interview, considering the activity range of the most common waterfowl species observed in Japan that are potential hosts for HPAIV [10]. A maximum of two control farms were selected for each case farm. If no farms within a 5 km radius satisfied the criteria, no control farms were selected. Farms officially identified as epidemiologically related farms for any case farm were excluded from the control group, because all birds in those farms were subjected to preventive culling without testing as soon as they were identified as epidemiologically related farms; thus, their infection status could not be determined conclusively.

### 2.2. Data Collection

A structured questionnaire was developed to collect the detailed data. As some farms operated multiple barns with varying features, data were collected separately for each barn. The questionnaire was divided into two sections: one covering information about the entire farm (“farm-level”) and the other covering details specific to each barn (“barn-level”). The farm-level section included 31 questions: seven on general farm information, nine on farm facilities and general poultry management, eight on hygiene practices upon entry into the premises, and seven on wildlife management on farms. The barn-level section consisted of 33 questions: 12 on barn structure and equipment, six on poultry management within the barn, five on hygiene practices upon entry into the barn, and 10 on wildlife management in and around the barn. The questionnaire was developed based on tools previously used in epidemiological investigations during disease outbreaks in Japan, including survey instruments from earlier case–control studies on foot and mouth disease in 2010 [23] and low-pathogenic avian influenza H5N2 in 2005 [24]. The complete questionnaire is provided in the Supporting Information S1.

Data from the control farms were collected through interviews with farm managers using a prepared questionnaire. The interviews were conducted in farm offices outside the farms, in nearby meeting rooms designated by farm managers, or via virtual meetings to ensure biosecurity of the farms. Prior to the interview, the purpose of the interview and information handling policies were explained to the participants, and their consent to participate in the study was obtained. Interviewers were selected from among the members of the Epidemiology Group of the National Institute of Animal Health. Each interview was conducted by at least two interviewers, who independently recorded their responses and cross-checked their records for consistency. In cases of discrepancies, the interviewers reconfirmed the responses with the farm managers via email or telephone. For the case farms, the corresponding data were extracted from the epidemiological on-site investigation reports prepared by a response team composed of officials from the Ministry of Agriculture, Forestry and Fisheries, experts from the National Institute of Animal Health, and prefectural animal health officials. These investigations were conducted immediately after HPAI was confirmed. Items that were not recorded or were unclear in the original investigation documents were marked as “no response.” Only poultry barns in which the outbreak was first detected were included in the study for each case farm. Conversely, for the control farms, all poultry barns on the premises were included, and data were collected for each barn. The reference time point for data collection was set to immediately prior to the outbreak for the case farms and to within 1 month before the corresponding outbreak for the control farms.

Responses to the questionnaire were entered into Microsoft Forms (Microsoft Corporation, Redmond, WA, USA) and exported to an Excel spreadsheet (Microsoft Corporation). Certain responses were dichotomized for further analysis. For example, the question on manure disposal offered three options: piling it up in a manure shed on the premises, processing it in a manure fermentation tank or incineration facility on the premises, or shipping it out of the premises without treatment. During data entry, these responses were recoded into a binary variable, “manure processed on the premises,” with response options of “yes” or “no.”

The distance from each farm to the nearest water source was determined using the method described in a previous study [18]. Aerial photographs from Google Earth Pro (Google LLC, Mountain View, CA, USA) were used to identify nearby ponds, lakes, and rivers. The straight-line distance from the edge of the nearest poultry barn to these water bodies was measured using the ruler function in Google Earth Pro. If water sources could not be clearly identified using Google Earth Pro alone, additional confirmation was made using Google Maps (Google LLC) and maps provided by the Geospatial Information Authority of Japan (https://maps.gsi.go.jp/). For farms where no water body was found within a 2 km radius, the nearest water source was recorded as “not identified.”

### 2.3. Statistical Analysis

Univariable analysis was performed to assess the association between farm-level and barn-level factors and HPAI occurrence. For farm-level questions, continuous variables, such as the number of laying hens, were converted into binary variables using their median values as cut-off points. Fisher's exact test was used to evaluate the association between each farm-level variable and HPAI occurrence. Variables with *p* < 0.1 were selected as candidates for multivariable analysis.

As selecting a specific barn from farms with multiple barns was not feasible, one barn was randomly selected per control farm, whereas the initially affected barn was used for each case farm. This resampling procedure was repeated 1000 times to generate 1000 datasets for barn-level analysis to ensure Monte Carlo credibility. Continuous variables were converted into binary variables using their median values as cut-off points, and Fisher's exact test was performed for each dataset. Variables with *p* < 0.1 in more than 500 of the 1000 trials were selected as candidates for the multivariable analysis. For the candidate variables, relationships between candidate variables were further assessed using Fisher's exact tests, considering biological and contextual relevance to identify variables to be included in the subsequent analysis.

Following the univariable analysis, a multivariable analysis was conducted using a generalized linear mixed model to account for regional variability in HPAI infection risk. The model included “outbreak area” as a random effect, defined as a 5 km radius around each case farm. When the outbreak areas of different case farms overlapped, they were combined into a single area for analysis. For the multivariable analysis, one poultry barn was randomly selected from each control farm across 1000 iterations, generating 1000 datasets. For each dataset, a generalized linear mixed model was constructed with HPAI occurrence as the response variable. Explanatory variables included farm- and barn-level factors selected based on the result of the univariable analysis, with outbreak area as a random effect. All possible combinations of explanatory variables were tested, and the model with the lowest Akaike information criterion (AIC) was identified. The model selected most frequently across 1000 trials was designated as the best model. Q–Q plots were generated to evaluate the model's fit. To estimate the confidence intervals (CIs) for the parameter coefficients, a new set of 1000 datasets was generated by randomly selecting one poultry barn from each farm. Logistic regression was conducted using the previously identified best model. In this analysis, CIs were constructed using the percentile method, in accordance with the approach used for bootstrap percentile CIs [25], with the bounds as the 2.5th and 97.5th percentiles of the simulated distributions. The CIs for each coefficient and odds ratio (OR) were calculated across 1000 trials.

All statistical analyses were performed using R (version 4.3.3; R Core Team, 2024). The following R packages were used for the analysis: car [26], performance [27], glmmML [28], glmmTMB [29], DHARMa [30], and MuMIn [31]. Additional utility packages (tidyverse, parallel, and openxlsx) were also employed for data handling and output generation.

## 3. Results

**Figure 1:**
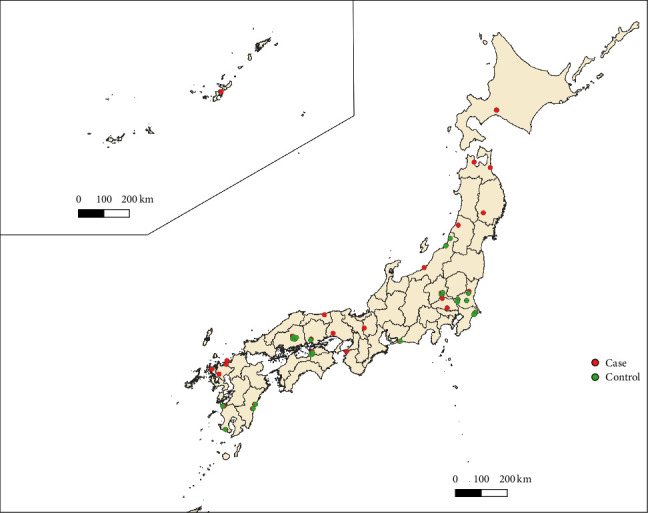
Locations of the farms included in the study. The map shows Japan and its prefectures. Okinawa Prefecture, which consists of small islands located far southwest of the main islands, is displayed as an inset in the upper-left corner at a different scale. The red dots indicate the locations of the case farms, representing all highly pathogenic avian influenza (HPAI)-affected commercial layer farms during the 2022–2023 season. The green dots indicate the locations of the control farms, which were located within a 5 km radius of the corresponding case farms.

Of the 84 cases of HPAI reported in poultry during the 2022–2023 season, 58 occurred on commercial layer farms across 22 prefectures. These 58 farms were designated as case farms for this study, and commercial layer farms located within a 5 km radius of each farm were considered candidate control farms. No eligible control farms were identified for 19 of the 58 cases, as no uninfected commercial layer farms meeting the criteria existed within a 5 km radius. Candidate control farms were identified in 14 additional cases, but these farms did not provide consent to participate in the study. For the remaining 25 cases, at least one candidate farm consented to the study, resulting in the selection of 26 control farms across 10 prefectures (Figure 1). Face-to-face interviews were conducted for 24 control farms, either on-site at the farm office or at the nearest prefectural animal hygiene service center. For the remaining two farms, interviews were conducted online at the request of the farm managers. The overall median number of laying hens per farm was 981,000 (interquartile range [IQR]: 35,000–244,975), with median values of 75,100 (IQR: 13,375–107,250) for the control farms and 118,650 (IQR: 45,500–325,000) for the case farms. The median number of poultry barns per farm was 4 (IQR: 2–7), with control farms having a median of 3 (IQR: 2–5) and case farms having a median of 5 (IQR: 2–7).

### 3.1. Univariable Analysis

First, all questionnaire items were reviewed to assess their suitability for the analysis. Items for which responses were missing or not applicable in ≥20% of the farms (i.e., at least 12 case farms or six control farms) were excluded from the analysis. As a result, the following items were removed: age of the barn, installation of an air duct under the cages, location of the feed scaler, induced molting status during the study period, installation of shutters attached to the openings of the egg conveyors, and options to prevent the entry of wild animals via the manure conveyor. Furthermore, items related to hygiene measures for visitors at the farm entrance were excluded, as hygiene protocols varied depending on the visitor type. For example, contractors entering farms to collect spent hens are typically required to change their shoes and clothing, whereas truck drivers who collect eggs may only be required to change their shoes.

For the variable regarding a distance from the nearest water body, distances between 50 and 300 m at 50 m intervals were assessed as candidate distances, considering the result of the previous study [18]. Univariable analysis was conducted using each of these candidate distances as an explanatory variable, and the 150 m distance obtained the smallest AIC. Therefore, “water bodies located within 150 m of the barns” were used in the subsequent analysis.

Table 1 provides the results of the univariable analysis of the farm-level variables. Variables with *p* < 0.1 included a large number of laying hens on the farm (≥100,000 birds), multiple caretakers (≥4), presence of employee residences within the farm premises, employees changing clothes when entering the hygiene control zone, and entry of private vehicles into the hygiene control zone. Among these, multiple caretakers (≥4) and employees changing clothes when entering the hygiene control zone were both positively associated with a large number of laying hens on the farm (≥100,000 birds). In contrast, the presence of employee residences within farm premises and the entry of private vehicles into the hygiene control zone were both negatively associated with a large number of laying hens on the farm (≥100,000 birds). As all farm-level variables with *p* < 0.1 were associated with a large number of laying hens on the farm (≥100,000 birds), these variables were considered inappropriate to evaluate independently of farm size, and thus they were represented collectively with the variable “large number of laying hens on the farm (≥100,000 birds).” Therefore, this was the only farm-level variable selected for inclusion in the multivariable analysis.

Table 2 shows the results of the univariable analysis of the barn-level variables. Univariable analysis was performed on all 1000 generated datasets, and the following variables yielded *p* < 0.1 in more than 500 iterations: a large number of birds per barn (≥30,000 birds), a large number of birds per cage (≥6 birds), vertically lined cages, a large number of cage tiers (≥5), closed-type house, a single-story barn, mechanical ventilation, automatic feeders, manure removal by conveyor, employees changing shoes at the barn entrance, employees changing shoes at the back door or avoiding the use of the back door, and proper repair of damaged barn walls and ceilings. Among these, several variables, including a large number of birds per cage, vertically lined cages, a large number of cage tiers, a single-story barn, mechanical ventilation, automatic feeders, and manure removal by conveyor, were significantly associated with closed-type house (all *p* < 0.001; Table 3). These variables were considered the structural characteristics of closed-type houses and were therefore represented collectively by the variable “closed-type house” in subsequent analyses. The barn-level variables selected for multivariable analysis were as follows: a large number of birds per barn (≥30,000 birds), closed-type house, employees changing shoes at the barn entrance, employees changing shoes at the back door or avoiding the use of the back door, and proper repair of damaged barn walls and ceilings.

### 3.2. Multivariable Analysis

Multivariable analysis was conducted to examine the association between HPAI occurrence and the following six candidate variables: a large number of laying hens on the farm (≥100,000 birds), a large number of birds per barn (≥30,000 birds), closed-type house, employees changing shoes at the barn entrance, employees changing shoes at the back door or avoiding the use of the back door, and proper repair of damaged barn walls and ceilings. The outbreak area was included as a random-effect variable. Each of the 1000 datasets was used to identify the model with the lowest AIC by generating models with all possible combinations of variables.  Table 4 lists the combinations of variables included in the models with the lowest AIC values and their frequencies across 1000 iterations. The model that most frequently had the lowest AIC, appearing in 526 of the 1000 datasets, included the following variables: a large number of laying hens on the farm (≥100,000 birds), employees changing shoes at the barn entrance, and employees changing shoes at the back door or avoiding the use of the back door. Therefore, the model incorporating these factors as explanatory variables and the outbreak area as a random effect was selected as the best model.

For each of the 1000 datasets generated by randomly selecting one poultry barn from each farm, logistic regression was performed using the best model, and ORs were calculated from the estimated coefficients (Table 5). The 95th percentiles of the ORs were used to determine the 95% CIs. The median OR for the variable “large number of laying hens on the farm (≥100,000 birds)” was 5.01, with a 95% CI of 4.15–5.87; the *p*-values for this coefficient were <0.05 in all 1000 trials. For the variable “employees changing shoes at the barn entrance,” the median OR was 0.061 (95% CI: 0–0.229), with *p*-values <0.05 in 954 out of 1000 trials. For the variable “employees changing shoes at the back door or avoiding use of the back door,” the median OR was 0.241 (95% CI: 0.240–0.313), with *p*-values <0.05 in 938 out of 1000 trials. To assess the model fit, Nagelkerke's pseudo *R*^2^ was calculated and ranged from 0.25 to 0.39 across the 1000 models. Residual diagnostics were performed using simulation-based methods. Three models with the highest, median, and lowest AIC were assessed as representative of 1000 models. Representative Q–Q plots and all diagnostic tests indicated no significant deviations from uniform distributions (*p*=0.921, 0.774, and 0.851, respectively), no evidence of dispersion (*p*=0.934, 0.998, and 0.996, respectively), and no excess outliers (*p*=1 for all three models) (Supporting Information S2).

## 4. Discussion

In this study, a large number of laying hens on the farm (≥100,000 birds) were identified as a potential risk factor for HPAI occurrence (OR 4.15–5.87), whereas employees changing shoes at the barn entrance (OR 0–0.229) and employees changing shoes at the back door or avoiding the use of the back door (OR 0.240–0.313) were identified as potential protective factors.

The identification of large-scale poultry farming as a risk factor aligns with the findings from a previous study on the 2020–2021 HPAI outbreak in Japan [10]. However, as the data were obtained from the national farm databases, which contain basic information such as flock size, poultry type, and farm location, the study did not include factors on poultry management practices, barn structures, or biosecurity measures and was, therefore, unable to determine whether the number of birds was a risk factor or whether associated management practices and housing structures contributed to the outbreak risk. During the 2022–2023 season, multiple outbreaks occurred in large commercial layer farms with modern, automated, and closed-type poultry barns, raising concerns about the role of barn structure and associated management practices in HPAI risk. Detailed information was collected from individual farms through interviews, allowing for the inclusion of structural and management-related variables in the analysis. The results indicated that closed-type housing did not significantly increase the risk of HPAI outbreaks, whereas the number of birds on farms was significantly associated with HPAI outbreaks. One possible explanation is that, assuming an equal risk for each bird, a larger flock size increases the probability of at least one bird becoming infected. Furthermore, large-scale farms may experience more traffic from vehicles and personnel, including those involved in transporting feed, pullets, and spent hens. These increased movements present more opportunities for viral introduction on farms.

Among the factors related to biosecurity measures, changing shoes at the barn entrance was identified as a potential protective factor. This finding is consistent with that of a case–control study on H5N6 HPAI outbreaks in egg-laying chicken farms in South Korea, which reported that changing shoes between chicken houses reduced the risk of infection [32]. These results suggest that changing shoes at the entrance of poultry barns can effectively reduce the risk of introducing HPAIV into poultry barns from the surrounding environment, which may be contaminated with wild bird feces and other contaminants. Changing shoes at the back door or avoiding the use of the back door was also identified as a protective factor. In addition to the main entrance, many barns have a back door that is primarily used to access equipment for manure removal. In this study, among the 68 farms that implemented shoe-changing protocols at the main barn entrance, 27 (39.7%) did not implement shoe-changing protocols at the back door. The back door is often used by workers responsible for manure disposal who do not handle poultry and typically enter only limited areas of the barn to operate machinery. As a result, compared to the main entrance used by workers handling live birds, biosecurity practices such as shoe-changing may have been inconsistently applied at the back door. In contrast, several farms with strong biosecurity awareness completely prohibited the use of the back door and maintained a strict separation between internal and external personnel to prevent cross-contamination. The findings of this study underscore the importance of reviewing and strengthening biosecurity measures, even on farms that already follow basic hygiene protocols, with particular attention paid to areas such as back door access.

## 5. Limitations

In this study, control farms were selected from nonoutbreak farms located within a 5 km radius of each outbreak farm, with a maximum of two farms selected per case. However, owing to factors such as the absence of nearby poultry farms, neighboring farms being classified as outbreak farms, or refusal of candidate farms to participate, only 26 control farms were included in the study. This limited sample size may have reduced the statistical power of the analysis, potentially obscuring true associations between exposures and outcomes. Some farms that declined to participate provided specific reasons, such as being in the midst of poultry house renovations in preparation for the upcoming HPAI season after experiencing intensive local HPAI outbreaks, which made scheduling interviews unfeasible. Others consistently refused to participate from the initial stage, without providing explicit reasons. The possibility that these nonparticipating farms may have shared certain characteristics (e.g., insufficient maintenance of structural damage to walls or ceilings) cannot be excluded, and such potential selection bias should be considered when interpreting the study's findings. Therefore, variables not retained in the final model or not found to be statistically significant in this study should not be interpreted as lacking relevance, as they may still represent risk or protective factors.

In addition, while data for the case farms were obtained from on-site investigations conducted immediately after the outbreak, data for the control farms were collected via interviews with farm managers several months after the HPAI season, without entering the premises for biosecurity considerations. Therefore, the evaluation of certain items, such as the proper repair of damaged barn walls and ceilings or the presence of wild animal traces, may have been more optimistic for the control farms than for the case farms. Moreover, while interviews at control farms were conducted in a standardized manner by a small group of trained personnel using a structured questionnaire, on-site investigations at case farms were conducted by multiple teams, without strictly adhering to the standardized questionnaire, which may have led to variability in data quality and recording. These limitations should also be considered when interpreting the findings of this study.

Furthermore, this study focused on H5N1 HPAI cases that occurred during the 2022–2023 winter season. Therefore, the findings may not be generalizable to outbreaks in other seasons or infections caused by different viral subtypes with varying pathogenicity or patterns of circulation in wild birds. To identify more broadly applicable risk factors, future studies should consider data spanning multiple HPAI seasons.

Despite these limitations, this study provides valuable insights by comparing outbreak and nonoutbreak farms during the largest recorded HPAI outbreak in Japan. The identification of potential risks and protective factors for infection is expected to improve preventive measures aimed at reducing HPAI outbreaks in poultry farms.

## 6. Conclusion

This case–control study identified a large number of laying hens on the farm as a potential risk factor for H5N1 HPAI outbreaks on commercial layer farms, whereas changing shoes at the barn entrance and implementing appropriate biosecurity practices at back doors were identified as potential protective factors. Given the continued risk of HPAIV introduction into Japan via migratory birds, strengthening farm-level biosecurity and husbandry practices based on epidemiological evidence is essential to prevent future HPAI outbreaks in poultry.

## Figures and Tables

**Table 1:** Univariable analysis results for farm-level variables.

Variables	Case farms (%)	Control farms (%)	*p*-Value	OR	(95% CI)
General farm information					
Large number of laying hens on farm (≥100,000 birds)	32 (55.2%)	7 (26.9%)	**0.019**	3.29	(1.11, 10.78)
Large number of employees (≥8)	34 (58.6%)	11 (42.3%)	0.237	1.92	(0.69, 5.51)
Multiple caretakers (≥4)	42 (72.4%)	12 (46.2%)	**0.027**	3.02	(1.05, 8.94)
Presence of unused barns	13 (22.4%)	3 (11.5%)	0.369	2.20	(0.53, 13.21)
Growing barns within premise	10 (17.2%)	3 (11.5%)	0.746	1.59	(0.36, 9.84)
Facilities and general poultry management					
Share employees with other farms	10 (17.2%)	8 (30.8%)	0.249	0.47	(0.14, 1.61)
Share facilities with other farms	25 (43.1%)	13 (50.0%)	0.638	0.76	(0.27, 2.13)
Share vehicles and equipment with other farms	9 (15.5%)	4 (15.4%)	1.000	1.01	(0.25, 4.98)
Presence of employee residences located within the farm premises	6 (10.3%)	7 (26.9%)	**0.098**	0.32	(0.08, 1.26)
Conduct induced molting	31 (64.6%)	17 (65.4%)	1.000	0.97	(0.31, 2.91)
Carcass disposed in the manure shed	13 (22.4%)	6 (23.1%)	1.000	0.96	(0.29, 3.55)
Share carcass storage with other farms	14 (24.1%)	7 (26.9%)	0.791	0.87	(0.27, 2.96)
Manure is processed within premise	45 (77.6%)	19 (73.1%)	0.782	1.27	(0.37, 4.11)
Share manure processing facility with other farms	22 (37.9%)	13 (50.0%)	0.344	0.61	(0.22, 1.73)
Sell composted manure on-site	7 (12.1%)	5 (19.2%)	0.501	0.58	(0.14, 2.59)
Sell table eggs on-site	1 (1.7%)	2 (7.7%)	0.225	0.21	(0.00, 4.31)
Hygiene practices upon entry into the premises					
Employees change clothes when entering hygiene control zone	49 (84.5%)	17 (65.4%)	**0.082**	2.84	(0.85, 9.65)
Employees change shoes when entering hygiene control zone	48 (82.8%)	22 (84.6%)	1.000	0.87	(0.18, 3.47)
Changing area located at the boundary of the hygiene control zone	32 (55.2%)	13 (50.0%)	0.813	1.23	(0.44, 3.44)
Entry of any visitors into barns	38 (65.5%)	17 (65.4%)	1.000	1.01	(0.33, 2.92)
Visiting vehicle other than transporters of feed, eggs, pullets, and spent hens enters hygiene control zone	37 (63.8%)	19 (73.1%)	0.461	0.65	(0.20, 1.97)
Private vehicle entering the hygiene control zone	16 (27.6%)	13 (50.0%)	**0.052**	0.39	(0.13, 1.11)
Vehicle disinfection gate installed	31 (53.4%)	14 (53.8%)	1.000	0.98	(0.35, 2.75)
Vehicle disinfection gate or high-pressure sprayer installed	50 (86.2%)	22 (84.6%)	1.000	1.13	(0.23, 4.80)
Disinfect vehicles entering hygiene control zone without exception	40 (69.0%)	22 (84.6%)	0.182	0.41	(0.09, 1.46)
Vehicle disinfection facility located boundary of the hygiene control zone	40 (69.0%)	20 (76.9%)	0.603	0.67	(0.19, 2.13)
Hygiene control zone is crossed by public road	12 (20.7%)	2 (7.7%)	0.208	3.09	(0.61, 30.72)
Wildlife management on farm					
Water body located on the premises	9 (15.5%)	5 (19.2%)	0.754	0.77	(0.20, 3.31)
Sightings of cats on the premises	36 (62.1%)	18 (69.2%)	0.626	0.73	(0.23, 2.14)
Sightings of cats and wildlife and their traces on the premises	50 (86.2%)	20 (76.9%)	0.347	1.86	(0.47, 7.05)
Sightings of ducks and other waterfowls on the premises	4 (6.9%)	2 (7.7%)	1.000	0.89	(0.12, 10.47)
Sightings of crows on the premises	48 (82.8%)	20 (76.9%)	0.557	1.43	(0.38, 5.08)
Water body located within 150 m of the barns	34 (58.6%)	10 (38.5%)	0.103	2.24	(0.80, 6.58)

*Note:* Values represent the number of farms implementing each practice. The percentages in parentheses indicate the proportion of farms with practices relative to the total number of farms in each group. The relevant *p*-values were calculated using Fisher's exact test. Bold values indicate *p*-value <0.1.

Abbreviations: CI, confidence interval; OR, odds ratio.

**Table 2:** Univariable analysis results for barn-level variables.

Variables	Case farms (%)	Control farms (%)	*p*-Value	OR (95% CI)	Count *p* < 0.1
Barn structure and equipment					
Large number of birds per barn (≥30,000 birds)	32 (55%)	8 (31%)	0.058	2.74 (0.95, 8.52)	**721**
Large number of birds per cage (≥6 birds)	36 (64%)	7 (30%)	0.012	4.04 (1.31, 3.71)	**1000**
Vertically lined cages	41 (73%)	8 (33%)	0.001	5.33 (1.74, 7.71)	**1** **000**
Large number of cage tiers (≥5)	32 (57%)	9 (38%)	0.144	2.20 (0.75, 6.76)	**515**
Closed-type house	31 (53%)	8 (31%)	0.062	2.55 (0.89, 7.95)	**911**
Single-story barn	52 (90%)	18 (69%)	0.028	3.78 (1.00, 5.23)	**1** **000**
Mechanical ventilation	35 (60%)	9 (35%)	0.035	2.84 (1.00, 8.57)	**1** **000**
Air intake from monitor roof	12 (21%)	2 (8%)	0.208	3.09 (0.61, 30.72)	0
Draw air into room via inlet	5 (22%)	22 (38%)	0.129	2.54 (0.78, 9.89)	0
Automatic feeders	57 (98%)	20 (77%)	0.003	16.46 (1.83, 796.16)	**1** **000**
Disinfection of drinking water	45 (78%)	16 (62%)	0.185	2.14 (0.69, 6.57)	0
Poultry management within the barn					
All in/all out	49 (84%)	21 (81%)	0.754	1.29 (0.30, 4.94)	0
Manure removal by conveyor	41 (71%)	8 (31%)	0.001	5.30 (1.79, 17.07)	**1000**
Eggs collected by conveyor	49 (84%)	18 (69%)	0.143	2.39 (0.69, 8.26)	0
Hygiene practices upon entry into the barn					
Employees change clothes at the barn entrance	22 (38%)	13 (50%)	0.344	0.61 (0.22, 1.73)	0
Employees change shoes at the barn entrance	44 (76%)	24 (92%)	0.131	0.27 (0.03, 1.31)	**529**
Employees disinfect hands at the barn entrance	47 (81%)	23 (88%)	0.533	0.56 (0.09, 2.41)	0
Employees use footbaths at the barn entrance	51 (88%)	25 (96%)	0.425	0.29 (0.01, 2.50)	0
Employees change shoes at back door or avoiding use of the back door	30 (52%)	21 (81%)	0.015	0.26 (0.07, 0.83)	**1000**
Wildlife management within the barn					
Sightings of cats inside the barn	7 (12%)	3 (12%)	1.000	1.05 (0.22, 6.86)	0
Sightings of wild animals and their traces in the barn	9 (16%)	4 (15%)	1.000	1.01 (0.25, 4.98)	0
Sightings of wild birds in the barn	13 (22%)	5 (19%)	1.000	1.21 (0.35, 4.92)	0
Sightings of rodents in the barn	41 (71%)	17 (65%)	0.621	0.27 (0.41, 3.77)	0
Rodent control measures conducted	52 (90%)	24 (92%)	1.000	0.0.72 (0.07, 4.45)	0
Wire mesh with <2 cm mesh size installed	41 (71%)	22 (85%)	0.275	0.44 (0.10, 1.59)	0
Proper repair of damaged barn walls and ceilings	28 (48%)	17 (65%)	0.164	0.50 (0.17, 1.41)	**871**

*Note:* A total of 1000 datasets were generated by randomly selecting 1 barn per farm in each iteration 1000 times, and univariable analysis was conducted for each dataset. The values represent the number of farms that implemented each practice in the barn in one of the 1000 datasets. The percentages in parentheses indicate the proportion of farms with the practice relative to the total number of farms in each group. Relevant *p*-values were obtained using Fisher's exact tests. Count *p* < 0.1 indicates the number of trials in which the *p*-value for the corresponding variable was <0.1 within 1000 datasets. Bold values indicate that the values in the “Counts *p* < 0.1” exceed 500, meaning that more than 500 out of 1000 trials had *p* < 0.1.

Abbreviations: CI, confidence interval; OR, odds ratio.

**Table 3:** Association between house type (closed-type vs. open-sided) and poultry house structural variables.

Variables	Open-sided (%)	Closed-type (%)	*p*-Value	OR (95% CI)
Large number of birds per cage (≥6)	7 (6%)	58 (97%)	<0.001	384.48 (78.45, 4218.55)
Vertically lined cages	13 (12%)	0 (92%)	<0.001	85.39 (28.06, 323.58)
Large number of cage tiers (≥5)	8 (7%)	9 (91%)	<0.001	117.16 (37.53, 441.82)
Single-story barn	71 (60%)	65 (100%)	<0.001	NE (10.52, NE)
Mechanical ventilation	15 (13%)	65 (100%)	<0.001	NE (98.74, NE)
Automatic feeders	76 (64%)	65 (100%)	<0.001	NE (8.77, NE)
Manure removal by conveyor	16 (14%)	58 (89%)	<0.001	50.87 (19.12, 156.91)

*Note:* Values represent the number of barns with each specified structural feature for 1 of the 1000 generated datasets. Parentheses indicate the proportion of barns with specific features relative to the total number of barns in each group (open-sided vs. closed-type). Odds ratios (ORs) and confidence intervals (CIs) for variables with a 100% positivity rate in closed-type barns could not be estimated and were denoted as NE (not estimable).

**Table 4:** Variables included in the models with the lowest AIC values across 1000 datasets.

Model	Large number of laying hens on farm	Large number of birds per barn	Closed-type house	Change shoes at the barn entrance	Change shoes at back door	Properly repairing damaged walls and ceilings	Count
1	●			●	●		526
2		●		●	●	●	342
3	●	●		●	●	●	100
4		●			●	●	20
5	●			●	●	●	12

*Note:* Count indicates the number of trials in which the model containing the corresponding variables was selected as the model with the lowest AIC among the 1000 trials.

Abbreviation: AIC, Akaike information criterion.

**Table 5:** Final results of the multivariable analysis.

Variables	OR	(95% CI)		Count *p* < 0.05
Large number of laying hens on farm (≥100,000 birds)	5.01	(4.15, 5.87)		1000
Employees change shoes at the barn entrance	0.061	(0.00, 0.23)		954
Employees change shoes at the back door or avoid using back door of the barn	0.241	(0.24, 0.31)		938

*Note:* Odds ratios (ORs) and 95% confidence intervals (CIs) were derived from the estimates of 1000 logistic regression trials. Count *p* < 0.05 indicates the number of trials in which the *p*-value for the corresponding variable was <0.05.

## Data Availability

Farm-level data, including responses to the questionnaire, are not publicly available because of personal data protection considerations in accordance with the Act on the Protection of Personal Information, Japan (https://www.ppc.go.jp/en/legal/).

## Supporting Information

Supporting Information 1 S1: Questionnaire used to collect farm- and barn-level information.

Supporting Information 2 S2: Q–Q plots for scaled residual for representative models.
