# Supplementary material for: Risk Factors for Introduction of H5N1 Highly Pathogenic Avian Influenza Virus in Japanese Commercial Layer Farms During the 2022–2023 Epidemic: A Case–Control Study
Source: Transbound Emerg Dis. 2025 Nov 12;2025:2658633. doi: 10.1155/tbed/2658633 (PMC12629688; doi:10.1155/tbed/2658633)
Supplement: Supporting Information 1 — S1: Questionnaire used to collect farm- and barn-level information. [file 2658633.f1.pdf]

Questionnaire for case-control study on HPAI risks on layer farms

Preface

This survey is being conducted by the National Institute of Animal Health of the National Agriculture and Food Research Organization (NARO) with the cooperation of prefectural governments for the purpose of conducting a case-control study on the risk of highly pathogenic avian influenza outbreaks.

This study aims to identify the factors associated with the risk of highly pathogenic avian influenza outbreaks and to consider more effective countermeasures. To achieve this, it is important to compare animal husbandry practices, including hygiene management, between affected and unaffected farms. Although these events occurred a while ago, please look back on your records and memories and answer as accurately as possible. Personal information and the names of related parties obtained through this survey will not be disclosed.

Additionally, for the survey, please prepare a site plan of the farm, such as all poultry barns with barn numbers, farm entrances and exits, manure processing facilities and compost sheds, dead bird storage and processing facilities, GP/FP facilities, vehicle disinfection equipment, offices, changing rooms, parking lots, water sources within the farm premises, direct sales locations for eggs and compost within the farm premises, fences surrounding the farm, and boundaries of the hygiene control area. Furthermore, if you could also prepare architectural drawings illustrating the structure of the barns, this would be helpful for the survey.

Target study period

Please answer based on the situation during the month prior to

**Farm name**	**Respondant**
	**name**<br>**title** ( )

Address

**Date of interview**	**Name(s) of the interviewer**
.	

Farm level questions

1 General information about the farm

Q1 Fill in the number of birds.

Egg laying hen（ ） Pullet or chicks（ ）

Q2 Fill in the number of barns at the study period.

Egg collection barns（ ） # of unoccupied barns（ ）
Grower barns （ ） # of unoccupied barns（ ）
Brooder barns （ ） # of unoccupied barns （ ）

Note: One building is considered one barn. The old barn that are no longer used as poultry barns in the current production cycle due to the downsizing of the business, should be exluded from the count.

Q3 Are there any abondoned poultry barns on the premises?

Yes ／ No

Note: This refers to unused barn other than those described in Question 2, "unoccupied barns." It includes barns where production equipment such as cages remains, but which have not been used for a long period of time due to downsizing or aging of facilities. It does not include cases where the equipment has been removed and the buildings have been converted into warehouses or other facilities.

Q4 Fill in the number of employees.

Permanent （ ） of whom poultry caretaker （ ）
Part-time （ ） of whom poultry caretaker（ ）
Foreign technical trainee（ ） of whom poultry caretaker（ ）

Q5 Do you share employees, facilities, and equipment/vehicles with other farms?

Yes ／ No
（employees facilities equipment/vehicle ）

Q6 (In case Q5 is yes,) do you share employees who work inside hygiene control zone with other farms?

Yes, on daily basis ／ Yes, but sometimes ／ Never
In which situation you share employees with other farms?
（ ）

Q7 Are there emloyees' residence on the premises?

Yes, inside hygiene controled zone ／
Yes, located at the boundary of hygiene controled zone／
Yes, located at other parts of the premises ／ No

Note: If the residence was located at the boundary and the employees have access to the hygiene control area via the residence, select " located at the boundary of hygiene control area". If the residence does not have access to the hygiene control area, select "located at other parts of the premises" even if the residence is physically located at the boundary.

2 Farm facility and general poultry management

Q1 Does the farm conduct induced molting as a part of routine husbandry practices?

Yes ／ No

Q2 Do you introduce pullets from external farms? Fill in the age for placement of the pullets into the egg collection barn.

From external source ／ From on-site grower barns<br>(age of the placement of pullet into egg collection barn days)

Q3 Average age of termination of egg laying hens (i.e. removal as spent hens).

(days)

Q4 How do you dispose poultry manure?

Dump into manure shed on the premises/<br>Load into the manure fermentation tank on the premises/<br>Collected by contractors/<br>Shipping out from the premises

Q5 Do you share a carcass storage or carcass processing equipment with other farms?

Sharing carcass storage etc. located on the premises/<br>Using carcass storage etc. in other farms /<br>Using shared storage or processing facility placed outside farm/<br>Carcass storage etc. not shared with other farms

Q6 How do you process poultry manure disposed from the poultry barns?

Piling up at manure shed on the premises/<br>Processing in manure fermentation tank or incineration facility on the premises /<br>Shipping out from the premises without treatrment

Q7 Do you share manure processing facilities (e.g., manure shed, fermentation tank) with other farms?

Sharing own facility with other farms/ Using facility in other farms/<br>Using shared manure composting facility placed outside farm/<br>Not sharing manure composting faclitity with other farms

Q8 Do you sell fermented manure fertilizer directly to the crop farms on-site? (i.e. customer visits the premises to purchase manure fertilizer)

Yes ／ No

Q9 Do you sell table eggs directly to the customers on-site? (i.e. customer visits the premises to purchase table eggs)

Yes ／ No

3 Hygiene practices upon entry to the premises

Q1 Select all hygiene measures carried out by employees upon entry to the premises.

Change clothes/ Change shoes/ Hand sanitation or use of dedicated gloves/ Footbath / Shower–in (all employees)/ Shower–in (poultry caretakers only)

Q2 Select all hygiene measures carried out by visitors upon entry to the premises.

Change clothes/ Change shoes/ Hand sanitation or use of dedicated gloves/ Footbath / Shower–in(all visitors) / Shower–in (visitors entering poultry barns)

Q3 Frequency of refreshing disinfectant solution of footbaths at the entrance.

Everyday / 3 times a week /Once a week / Less than once a week / No footbath

Q4 Select all visitors potentially entered poultry barns.

Veterinarians / Support staff from other farms / Staff for placement of pullets / Staff for removal of spent hen / Constraction workers / Others

Q5 Select all vehicles potentially entered farm premise

Feed truck / Egg collection vehicle / Carcass collection vehicle/<br>Pullets transporting vehicle /Spent hen transporting vehicle /<br>Compost purchasers' vehicle / Compost shipment vehicle /<br>Manure delivery vehicle / Egg purchasers' vehicle / Veterinarians vehicle /<br>Private vehicle including employees' commuter vehicle /<br>Construction vehicle

Q6 Select all facilities and equipment for vehicle disinfection on the premises.

Disinfection gate / Pressure sprayer / Small sprayer / Slaked lime

Q7 Select target vehicles for disinfection from below.

All vehicles entering hygiene control zone /<br>Some exceptions /<br>No vehicle disinfection practiced

Q8 Indicate the location of the vehicle disinfection facility or equipment.

Boundary of hygiene control zone / Inside hygiene control zone / Outside hygiene control zone / No vehicle disinfection practiced

Note: Select "Boundary of hygiene control zone" if the vehicle enter the hygiene control zone via disinfection facility. Select "Inside hygiene control zone" if the vehicle receive disinfection at the disinfection station placed inside the hygiene control zone. Select "Outside hygiene control zone" if the vehicle enter the hygiene control zone via non-hygiene control area zone disinfection.

4 Wildlife management on farm

Q1 Are there any water sources which is filled with water throughout winter (e.g., ponds, swamps, waterways, water pools without covering) on the premises?

Yes ／ No

Q2 Do you sight cats on the premises? If yes, indicate the locations.

Often / Sometimes / Rarely / Never<br><br>(near poultry barns / manure storage /<br>near watersource / near egg collection facility)

Note: "often" indicates once a week and more, "sometimes" indicates one or two times a months, "rarely" indicates once in a several months. These definitions are also applied to the following questions.

Q3 Do you site medium-sized wild mammals such as weasels, foxes and racoons on the premises? If yes, indicate the locations.

Often / Sometimes / Rarely / Never<br><br>(near poultry barns / manure storage /<br>near watersource / near egg collection facility)

Q4 Do you observe traces of medium-sized wild mammals and cats on the premises (e.g., feces, bite marks) ? If yes, indicate the locations.

Often / Sometimes / Rarely / Never<br><br>(near poultry barns / manure storage /<br>near watersource / near egg collection facility)

Q5 Do you sight waterfowl such as ducks on the premises? If yes, indicate the locations.

Often / Sometimes / Rarely / Never<br><br>(near poultry barns / manure storage /<br>near watersource / near egg collection facility)

Q6 Do you sight crows on the premises? If yes, indicate the locations.

Often / Sometimes / Rarely / Never<br><br>(near poultry barns / manure storage /<br>near watersource / near egg collection facility)

Q7 If you select "Often" or "Sometimes" in Q6, select the state of crow flocks you observe.

Large flock with dozens of crows /<br>Small flock with less than 10 birds /<br>Pair or solitary

That is all for farm level questions.
Questions regarding each barn will follow.

Barn level questions

Barn No.

Note: Fill in the questionnaire for each barn. However, if there are duplicate responses regarding poultry barn structure or husbandry practices across multiple poultry barns, you may wrap the responses in one questionnare by listing the barn numbers.

1. Barn structures and equipment

Q1 Fill in the age of the barn.

Approx. years old (If major renovations have been carried out, the number of years since renovation)

Q2 Fill in the number of birds per barn / number of birds per cage / number of rows

# of birds per barn # of birds per cage

(H-type cage tiers／ A-type cage tiers／ free-range)

Q3 Select the barn type.

(Closed-type house ／ Open-sided house)
(Single-story ／ Two-stories)

Note: Even if the barn window was covered with a curtain at the time of the interview, select "open-sided house" if the house allows light and air to enter by opening the curtain.
Note: The term "two stories means" that manure is deposited on the ground floor while birds are kept at upper floor. Select "single story" for H-type cages with multiple tiers, even if the grating platform is installed every few tiers.

Q4 Select the ventilation system during the study period.

Mechanical ventilation
(positive pressure ／negative pressure ／tunnel ventilation)
Natural ventilation ／
Unknown

Q5 Indicate location of air inlet or fan which supplies air into barn from outside.

Monitor roof / Upper side other than monitor roof ／ Side of the barn

Bottom of the barn ／ Side open window ／ Unknown

Q6 Indicate location of air inlet which supplies air into the bird occupied area.

Ceilings ／ Side of the barn ／ Bottom of the barn ／
No inlet ／ Unknown

Q7 Indicate location of exhausting fan if installed.

Upper / Gable end / Side / No fan

Q8 Is the air duct installed under cages?

Yes ／ No

(If yes, air supplied by the duct is) external air ／ air circulating in the barn

Q9 Is the automatic feeder installed?

Yes ／ No(i.e. feed maually using scoops)

Q10 Indicate location of the feed scaler.

Under the feed tank / Outside barn or in the dedicated room /<br>Inside poultry barn / Unknown

Q11 Indicate type of water to be supplied to poultry.

Tap water ／<br>Ground water(with disinfectant / without disinfectant)／<br>River water(with disinfectant / without disinfectant)

Q12 Indicate water supply method.

Nipples ／ Gutter

2 Poultry management within the barn

Q1 Do you implement all-in/all-out ?

Yes ／ No

Q2 Indicate inducing molting status during the study period.

On-going ／ Within a month after completion of molting／<br><br>None of them ／ Unknown

Q3 Indicate manure removal method from the barn.

Manure conveyor / Remove from barn floor using wheel loader /<br>Remove from underfloor pit by wheel loader

Q4 Indicate frequency of manure removal from the barn.

Daily / A couple of times a week / At the time of all-out only

Q5 Indicate egg collection and egg trasnportation method.

Transport collected eggs to the internal egg collection facility by conveyor /<br>Transport collected eggs to the external egg collection facility by conveyor /<br>Transport collected eggs to the internal egg collection facility without using conveyor /<br>Directly shipping out from the barn

Q6 Indicate frequency of disinfection around the poultry barn.

Slaked lime<br>(3 times a week and more / once a week / 1 to 2 times a month / less)<br><br>Disinfectant<br>(3 times a week and more / once a week / 1 to 2 times a month / less)

3 Hygiene practices upon entry to the barn

Q1 Select all hygiene measures carried out by employees upon entry to the poultry barn from main barn entrance.

Change clothes/ Change shoes/<br>Hand sanitation or use of dedicated gloves / Footbath

Q2 Select all hygiene measures carried out by employees when entering the barn from back door.

Change clothes/ Change shoes/ Hand sanitation or use of dedicated gloves/<br>Footbath / Never using back door / None of them

Note: Backdoor means the door which is not used as main entrance by poultry caretakers, but used by manure conveyer operators who temporarily enter the barn during operation or used to deliver materials into barns.

Q3 Select all hygiene measures carried out by visitors upon entry to the poultry barn.

Change clothes/ Change shoes/<br>Hand sanitation or use of dedicated groves/ Footbath

Q4 Indicate frequency of refreshing disinfectant solution of footbath a the barn entrance.

Everyday / 3 times a week /Once a week /<br>Less than once a week / No footbath

Q5 Select the follwing options best illustrates the situation regarding the layout of the boots changing area.

The location for changing boots is clearly delineated, and the boots before and after changing do not come into contact with each other/<br>The location for changing boots is not clearly delineated so that boots before and after changing may come into contact with each other/<br>Changing boots is not practiced

4 Wildlife management within in and around the barn

Q1 Do you sight cats in the barn?

Often / Sometimes / Rarely / Never

Q2 Do you site medium-sized wild mammals such as weasels, foxes and racoons in the barn?

Often / Sometimes / Rarely / Never

Q3 Do you observe traces of medium-sized wild mammals and cats in the barn (e.g., feces, bite marks) ?

Often / Sometimes / Rarely / Never

Q4 Do you sight crows and their traces in the barn (e.g., feathers, feces) ?

Often / Sometimes / Rarely / Never

Q5 Do you sight rodents and their traces in the barn?

Often / Sometimes / Rarely / Never

Q6 Select all rodents control activities conducted in the barn.

Contract a professional pest control service /
Pest control carried out by employees /
No activities conducted

Q7 Select the following option best illustrates the status of wire mesh installation at the openings and windows of the barn.

Wire mesh with the mesh size 2 cm or less is installed /
Wire mesh with the mesh size larger than 2 cm is installed /
No wire mesh installed

Q8 Select the following option best illustrates maintenance status against damages and gaps of the barn.

Always monitored and damages properly repaired/
Regularly monitored but not all damages are repaired /
Observe significant damages which have not been repaired

Q9 Are there shutters attached to the openings for the egg conveyor to prevent the entry of wild animals?

Shutters installed at all openings ／ Shutters partially installed ／

Shutters not installed ／ Egg conveyors not installed

Q10 Select all options to prevent entry of wild animals via manure conveyer.

Cover openings of the pit when not is use ／ Install shutter at the end of conveyor

Cover all openings by wire net or coverings ／ Conveyor not intalled
