# Supplementary material for: Risk Factors for Introduction of H5N1 Highly Pathogenic Avian Influenza Virus in Japanese Commercial Layer Farms During the 2022–2023 Epidemic: A Case–Control Study
Source: Transbound Emerg Dis. 2025 Nov 12;2025:2658633. doi: 10.1155/tbed/2658633 (PMC12629688; doi:10.1155/tbed/2658633)
Supplement: Supporting Information 2 — S2: Q–Q plots for scaled residual for representative models. [file 2658633.f2.docx]

**Supplemental figure 1.** Quantile–quantile (QQ) plot of scaled residuals for the model with the lowest AIC. The plot compares the observed and expected residual quantiles to evaluate model fit. The residuals align closely with the theoretical quantiles. No significant deviation was detected using the Kolmogorov–Smirnov test (p = 0.851), dispersion test (p = 0.996), or outlier test (p = 1.000), indicating no evidence of non-uniformity, over/under-dispersion, or extreme residuals.

**Supplemental figure 2.** QQ plot of scaled residuals for the model with median AIC. The plot compares the observed and expected residual quantiles to evaluate the model fit. The residuals align closely with the theoretical quantiles. No significant deviation was detected by the Kolmogorov–Smirnov test (p = 0.774), dispersion test (p = 0.996), or outlier test (p = 1.000), indicating no evidence of non-uniformity, over/under-dispersion, or extreme residuals.

**Supplemental figure3.** QQ plot of the scaled residuals for the model with the highest AIC. The plot compares the observed and expected residual quantiles to evaluate the model fit. The residuals align closely with the theoretical quantiles. No significant deviation was detected using the Kolmogorov–Smirnov test (p = 0.921), dispersion test (p = 0.934), or outlier test (p = 1.000), suggesting no evidence of non-uniformity, over/under- dispersion, or extreme residuals, respectively.
